# Far Transfer Effects of Trainings on Executive Functions in Neurodevelopmental Disorders: A Systematic Review and Metanalysis

**DOI:** 10.1007/s11065-022-09574-z

**Published:** 2023-01-12

**Authors:** Clara Bombonato, Benedetta Del Lucchese, Costanza Ruffini, Maria Chiara Di Lieto, Paola Brovedani, Giuseppina Sgandurra, Giovanni Cioni, Chiara Pecini

**Affiliations:** 1 Department of Developmental Neuroscience, IRCCS Stella Maris Foundation, Calambrone, Pisa, Italy; 2 Tuscan Programme of Neuroscience, University of Florence, Pisa and Siena, Italy; 3 Department of Education, Intercultures, Literatures and Psychology (FORLIPSI), University of Florence, Languages, Florence, Italy; 4 Department of Clinical and Experimental Medicine, University of Pisa, Pisa, Italy

**Keywords:** Executive functions, Neurodevelopmental disorders, Far effect, Children

## Abstract

Executive Functions are a set of interrelated, top-down processes essential for adaptive goal-directed behaviour, frequently impaired across different neurodevelopmental disorders with variable degrees of severity. Many executive-function-training studies in children with neurodevelopmental disorders have focused on near effects, investigating post-treatment improvements on directly trained processes, while enhancements of skills not directly trained, defined as far effects, are less considered, albeit these could be extremely relevant for reducing the negative impact of a disorder’s core symptomatology. This systematic review and metanalysis aims to investigate the far effect outcomes after EF training in children with different types of neurodevelopmental disorders. 17 studies met the inclusion criteria for the systematic review, while 15 studies were selected in the metanalysis. An overall statistically significant effect size was found in the majority of far effect outcome measures considered in the studies. In particular, trainings on executive functions determine significant far effects on daily life functioning (0.46, 95% CI: [0.05–0.87]) and clinical symptoms (0.33, 95% CI: [0.15–0.51]). Despite a high variability of the results, intensity, frequency and the laboratory/life contexts dimension seem to be the most influential variables in determining far effects. This systematic review and metanalysis highlights the need to measure far effects of executive function training in neurodevelopmental disorders, selecting treatments not only on directly targeted processes, but also according to far impacts on the functional weakness of the disorder.

## Introduction

### Executive Functions: Definition

Executive Functions (EFs) represent a complex cognitive domain consisting of a set of top-down functions essential for adaptive goal-directed behaviour (Lehto et al., 2003; Miyake et al., 2000). EFs allow to formulate, plan, and organize ideas, cope with challenges and novelties, resist temptations and stay focused (Diamond, 2013). There is an ongoing debate as to the extent to which EFs can be fractionated or be unified into a single concept, both in adults and during development (for example, Morra et al., 2018). The model that may best explain executive functioning during development has been put forward by Adele Diamond (Diamond, 2013; Diamond & Ling, 2016), based on the conceptualizations of Miyake and colleagues (Friedman & Miyake, 2017; Miyake et al., 2000). Three early and distinct, although interrelated, components are identified in this model: inhibition, working memory and cognitive flexibility, whose interaction allows for the development of higher order EFs such as reasoning, problem solving and planning.

Inhibitory control is the ability to voluntarily resist temptations and impulsive actions (i.e., response inhibition) and to maintain selective attention by suppressing non relevant information (i.e., interference control). Inhibitory control is a fundamental skill involved both in cognitive activities, such as abstract reasoning, and in affective and emotional challenges allowing for more appropriate behaviours geared to internal or external goals (Zelazo & Mller, 2002; Zelazo et al., 2005). Inhibitory control supports the development of self-regulation, which requires the ability to maintain optimal cognitive, emotional and motivational arousal levels.

Working memory is a complex and multi-component mental system where information can be temporarily stored. It refers to the ability to actively maintain, monitor, update and manipulate verbal or visual-spatial information (Baddeley, 2003; Baddeley & Hitch, 1994; Smith & Jonides, 1999).

Cognitive flexibility is the ability to shift among different tasks, rules or mental contents. It supports creative thinking and the capacity to solve problems in different ways or see things from different perspectives.

EFs develop from preschool-age to childhood and into adulthood (Hughes et al., 2009; Huizinga et al., 2006; Lehto et al., 2003; Somerville & Casey, 2010) following maturation of prefrontal circuitries and their connections (Gilbert & Burgess, 2008). A single-undifferentiated executive factor was found in younger children of preschool age (Wiebe et al., 2011), whereas two separate dimensions consisting of inhibition and working memory were identified in children older than 5 years of age (Lee et al., 2013; Miller et al., 2012; Usai et al., 2014). Cognitive flexibility emerges later in development (Lee et al., 2013; Lehto et al., 2003) after the inhibition and working memory abilities have been established. Subsequently, these three basic EF components support the emergence of more complex and high-level EFs, including abstract reasoning, problem solving and planning, also referred to as Fluid Intelligence (Collins & Koechlin, 2012; Diamond, 2013; Lunt et al., 2012).

EFs have also been differentiated into “cool” and “hot” processes (Zelazo & Carlson, 2012). The former domain, mainly subserved by the lateral prefrontal cortex, includes cognitive EF skills, elicited under relatively abstract, de-contextualized, non-affective conditions. Hot EF processes, mainly subserved by ventromedial prefrontal cortex and operating in motivationally and emotionally significant high-stakes situations, involve decision making, gratification delay and theory of mind (Wilson et al., 2018; Zelazo & Carlson, 2012).

In typically developing children, persistent difficulties affecting EFs, even if minor, represent a risk factor for development and can predict learning skills (Alloway & Alloway, 2010; Clark et al., 2014; LeFevre et al., 2013; Steele et al., 2012), academic achievement, job success, physical and mental wellbeing (McClelland et al., 2013; Moffitt et al., 2011; St Clair-Thompson & Gathercole, 2006).

## EFs and Neurodevelopmental Disorders

It is currently well accepted that EFs are frequently impaired across different developmental disorders (Bausela Herreras et al., 2019; Pennington & Ozonoff, 1996). In some neurodevelopmental disorders an EF deficit may be a part of the core cognitive symptoms, while in others, a weakness of EFs is associated with specific deficits and help to define different subtypes of the disorder. Finally, poor executive abilities could be due to the reduced efficiency of other cognitive and sensory-motor functions.

A deficit in inhibition, and in particular in the ability to inhibit responses, was described as one of the core deficit of Attention Deficit Hyperactivity Disorder (ADHD) (Barkley, 2006, 2018). According to Barkley, a deficit in inhibition may cause, in turn, deficits in working memory, emotional regulation, reconstitution and internalization of language, leading to difficulties in the self-regulation of social interaction. Indeed, in ADHD other EFs are also compromised, notably working memory, divided attention, cognitive flexibility, planning, sustained attention and theory of mind (reviews: Elosúa et al., 2017; Jiménez-Figueroa et al., 2017; Lambek et al., 2011; Mary et al., 2016; Molnar, 2007; Pineda-Alhucema et al., 2018; Sergeant et al., 2002; Willcutt et al., 2005). In many studies, also the hot components of EF are impaired in individuals with ADHD, for example delay aversion, Theory of Mind and decision-making (reviews and meta-analysis: Bora & Pantelis, 2016; Groen et al., 2013; Mowinckel et al., 2015; Patros et al., 2016; empirical studies: Braaten & Rosén, 2000; Yang et al., 2011). Individuals with Intellectual Disability (ID) display worse EFs abilities than subjects with the same chronological and mental age (review and meta-analysis: Hronis et al., 2017; Spaniol & Danielsson, 2019; Tungate & Conners, 2021; empirical studies: Costanzo et al., 2013; Danielsson et al., 2010; Carney et al., 2013).

Children with Developmental Coordination Disorder (DCD) present EFs impairment in several domains, such as working memory (especially visuospatial), inhibitory control, attention, flexibility and metacognitive aspects of action planning (reviews and metanalysis: Leonard et al., 2015; Wilson et al., 2013, 2017; empirical studies: Piek & Dyck, 2004; Sartori et al., 2020). Moreover, some evidence supports deficits in hot executive functions in children with DCD, as they have a high sensitivity to immediate gratification and to distracting emotional stimuli that underly low decision-making skills in emotionally activating situations (Rahimi-Golkhandan et al., 2014, 2015, 2016). Some difficulties in EFs remain distinctive features of individuals with DCD even in middle childhood, adolescence and early adulthood and limit children’s ability to improve automatic motor control and motor skills in daily activities (Bernardi et al., 2018; Wilson et al., 2017).

Executive functions are fundamental for cognitive-linguistic translation (Berninger et al., 2012), the basis for language learning (Arrington et al., 2014; Berninger et al., 2012; Swanson, 2000, 2006), and appear to be in a reciprocal and complex relationship with language development (Bishop et al., 2013). It is therefore understandable that individuals with Developmental Language Disorders (DLDs) show cognitive difficulties that are not limited to the language domain. In particular, this clinical population presents difficulties with multiple components of EFs (meta-analysis and review: Kapa & Plante, 2015; Pauls & Archibald, 2016; empirical study: Andrés-Roqueta et al., 2021; Henry et al., 2012; Roello et al., 2015) and related functions such as processing speed ( Miller et al., 2001), non-verbal reasoning (Gallinat & Spaulding, 2014), procedural memory (Lum et al., 2012), motor control (Finlay & McPhillips, 2013). The most compromised EFs in this disorder are inhibition (Marini et al., 2020; Pauls & Archibald, 2016), cognitive flexibility (Pauls & Archibald, 2016), working memory both phonological (Duinmeijer et al., 2012; Marini et al., 2014) and visuospatial (Vugs et al., 2013), updating (Marini et al., 2020) and attentional control in verbal and non-verbal tasks (Dispaldro et al., 2013; Duinmeijer et al., 2012; Ebert & Kohnert, 2011; Finneran et al., 2009; Montgomery, 2008; Montgomery et al., 2009; Spaulding et al., 2008). Learning to read, text comprehension and mathematical competences are linked to working memory, inhibition, cognitive flexibility, updating and attentional control and planning (Cartwright & Smith, 2017; Gilmore & Cragg, 2014; Zaccoletti & Mason, 2018).

Individuals with Specific Learning Disorder (SLD) are characterized by difficulties in executive functions domains such as planning, cognitive flexibility, verbal and visuospatial working memory, attentional control and inhibition (El Wafa et al., 2020; Schuchardt et al., 2008). Developmental Dyslexia is the most studied disorder in terms of executive dysfunctions. Impairments or weaknesses have been reported in visual-spatial (Altemeier et al., 2008; Helland & Asbjørnsen, 2000; Menghini et al., 2010) and auditory attention (Buchholz & McKone, 2004; Casco & Prunetti, 1996; Dufor et al., 2007; Facoetti et al., 2000; Valdois et al., 2004), shifting (Hari & Renvall, 2001; Laasonen et al., 2012), verbal categorical and phonological fluency, verbal and visual short-term memory, verbal and visual-spatial working memory (Varvara et al., 2014), inhibition of irrelevant information (Brosnan et al., 2002; Everatt et al., 1997; Reiter et al., 2005), maintaining relevant information in working memory (meta-analysis: (Booth et al., 2010). In particular, the working memory deficit is considered one of the major markers of Dyslexia, both in its verbal and visuospatial components (Bacon et al., 2013; Brosnan et al., 2002; Helland & Asbjrnsen, 2004; Martinussen & Tannock, 2006; Menghini et al., 2011; Poblano et al., 2000; Smith-Spark & Fisk, 2007; Swanson et al., 2009).

EFs have been found to be frequently impaired in children with Autism Spectrum Disorder (ASD), characterized by a deficit in cognitive flexibility, planning and inhibiting preponderant responses (Hill, 2004; Jiménez-Figueroa et al., 2017; Kenworthy et al., 2005; Landa & Goldberg, 2005; Lopez et al., 2005; Ozonoff et al., 1994; Rinehart et al., 2001; Robinson et al., 2009; Shu et al., 2001; Verté et al., 2005).

Finally, EFs are crucial for adaptive behaviour, in as much as efficient executive functioning during child development is able to predict health and well-being in adulthood (Moffitt et al., 2011). Considering that especially in childhood, EFs are indeed highly responsive to environmental influences (Jolles & Crone, 2012; Klingberg, 2010), it is important to identify early EF impairments in order to intervene and improve developmental trajectories.

### EF Interventions

Convergent evidence suggests that it is possible to improve EFs through cognitive training (Diamond & Lee, 2011) and some findings demonstrated a strengthening of the neural circuits underlying the trained EFs by intensive practice (Brehmer et al., 2011; Crespi et al., 2018; McNab et al., 2009; Rueda et al., 2012). Given the importance of EFs in development and their variability in the severity of their impairment in different neurodevelopmental disorders, many studies have analyzed the effectiveness of different approaches both for the enhancement of EFs and for the generalization effect on other cognitive and daily life functioning. Some key principles of clinical practice for an intervention to be helpful foresee contextual support and the use of compensatory aids, the use of problem-solving and metacognitive strategies aimed at improving specific task trained but also applicable to a variety of everyday situations (Krasny-Pacini et al., 2018).

Many types of EF intervention are reported in the literature: computerized training, non-computer games, physical activities, classroom curricula, art activities, mindfulness practices, and biofeedback. Computer-based programs, such as CogMed Working Memory Training (www.cogmed.com) and Braingame Brian (Prins et al., 2013), are among the most popular interventions used for the improvement of working memory and for the enhancement of inhibition and cognitive flexibility respectively. Evidence shows that although these treatments have a solid effect in improving the practiced skills, such as inhibition and working memory span (Beck et al., 2010; Chacko et al., 2014; Di Lieto et al., 2021; Gibson et al., 2011; Kidokoro et al., 2014; Klingberg et al., 2005; Løhaugen et al., 2011; Lundqvist et al., 2010; Melby-Lervåg et al., 2016), the improvements do not seem to transfer to untrained domains (Blair & Razza, 2007; Diamond, 2012; Diamond & Lee, 2011; Diamond & Ling, 2016), nor to untrained EF skills (Kassai et al., 2019), nor to everyday life contexts if the intervention is not included in these scenarios (Blair, 2017). The efficacy of EF treatments through physical activities (Best & Miller, 2010; Ng et al., 2017; Tomporowski et al., 2008) and non-computerized games (Tominey & McClelland, 2011) has also been demonstrated. The effectiveness of these interventions could depend on the activation of strategies and cognitive skills related to EFs. Furthermore, complex motor activity activates brain regions related to the prefrontal cortex which may produce immediate physiological responses (increased blood flow, oxygen and brain derived neurotrophic factor-BDNF) which in turn facilitate cognitive performance and learning (Best & Miller, 2010). The presence of cognitive challenges within physical activities requiring flexible adaptation of behaviour seems to produce greater effects on EFs than physical activities involving only aerobic components or automated motor responses (Best & Miller, 2010; Diamond, 2015). Other promising treatment approaches are classroom curricula specifically designed to promote EFs, such as Tools of the Mind (Bodrova & Leong, 2006). These approaches are inserted in the daily practice of children, facilitating the generalization of the skills learned and their application in new contexts. Furthermore, these programs do not require any specific materials, can be conducted in school by teachers and can include a large number of participants (Diamond & Lee, 2011). Not only specific curricula design to promote EF, but also some academic discipline as art activities (Diamond, 2012; Diamond & Lee, 2011; Diamond & Ling, 2016), such as music and drama, requiring inhibitory control and cognitive flexibility are able to produce benefits in EF skills (Schellenberg, 2004; Thibodeau et al., 2016). Another approach to foster children’s EFs is providing them with strategies of self-regulation, both through teaching skills targeting metacognitive intervention, useful for daily life challenges, and through mindfulness practises. This latter activity requires attention (Zelazo & Lyons, 2012) and self-control, reducing anxiety and stress, in the meanwhile, working both on a cognitive and emotional level (Zenner et al., 2014).

Finally, also biofeedback, a technique that uses the electroencephalographic (EEG) or electromyographic (EMG) signal for learning voluntary self-control of some psychophysiological processes that are usually involuntary, are effective on attention and self-regulation, fostering self-teaching strategies to control physiological reactions (Niv, 2013). Neurofeedback training has also been reported to be effective in reducing clinical symptoms in children and adolescents with ADHD (Arns et al., 2009). However, a more recent meta-analysis highlighted the lack of efficacy of neurofeedback treatment tested by standardized tests on EFs in ADHD children (Louthrenoo et al., 2022). This inconsistency in the literature evidence could be due to the different outcome measures considered.

Despite the wide amount of data supporting the usefulness of EF training, the characteristics that make an EF intervention effective are not fully understood. The review by Diamond and Ling (2016) highlights that interventions involving socio-emotional components and physical exercise have the greatest effectiveness, as long as cognitive challenges are included within the proposed activities. Moreover, the exercises must be calibrated on the subject's abilities, as to represent a challenge rather than only skill practice. Other variables influencing the success of the training are the personal characteristics of the person conducting the program and the starting impairment level of the participants, as it seems that greater benefits are observed in conditions of greater initial EF impairment. Furthermore, Blair (2017) emphasizes the importance of placing the intervention within an everyday life context in order to increase ecological validity and generalization. However, interventions on EFs must not become a burden for the family system, already challenged by child’s difficulties, but have to involve the caregivers in an appealing way, favouring skills acquisition useful to support daily life functioning (Krasny-Pacini et al., 2018).

Since EFs are highly correlated with other cognitive functions, their impairment can determine cascade effects on other neuropsychological processes. For this reason, EF improvements could produce effects on functions untrained but correlated with EFs, resulting in important benefits for children's daily functioning. These non-specific effects have been defined by the literature as far-transfer effects, i.e. effects of training on different processes correlated with practiced skills (Melby-Lervåg & Hulme, 2013; Sala & Gobet, 2016, 2017), as opposed to near transfers, i.e. post-treatment improvements in tasks that require directly trained processes (Kassai et al., 2019; Melby-Lervåg & Hulme, 2013; Sala & Gobet, 2016, 2017). Transfer has been defined not only in terms of improvements in different tasks, but also in terms of improvement along time intervals and contextual similarity, and in each of these dimensions the transfer can be near or far (Klahr & Chen, 2011). Linked to the conceptualization of transfer in terms of context dimension, Diamond and Ling (2016) analysed the narrow transfers, i.e., improvements of the abilities trained within the treatment but in other contexts where the same skills are required. The authors argue that “people improve on the skills they practice and that transfers to other contexts where those same skills are needed […]; improvement does not seem to transfer to other skills" (Diamond e Ling in Novick et al., 2020, pages 460–461). The question about the possibility of producing far transfer after EF training is still open, as pointed out by the review by Katz and Saha (2020) on children with developmental disorders (see Novick et al., 2020). Katz and Saha analysed many studies, showing the heterogeneity of results (Chooi & Thompson, 2012; Heinzel et al., 2014; Jaeggi et al., 2014; Kundu et al., 2013; Redick et al., 2013; Stephenson & Halpern, 2013; Thompson et al., 2013), varying from the absence of transfer effects (Melby-Lervåg & Hulme, 2013) to significant effects on skills far from those trained, as fluid intelligence (Au et al., 2015; Karbach & Verhaeghen, 2014). In order to disambiguate the question, it is necessary to develop and use dynamic outcome measures able to detect the effective EFs improvement after a treatment, as well as transfer effects on other processes, taking into account the ecological validity and the test–retest effect (Krasny-Pacini et al., 2018).

The present systematic review aims to investigate the presence of far-transfer effects following executive function training, limiting the analysis to children with neurodevelopmental disorders and considering as far-transfer effects any skill not directly trained by the treatment and assessed post intervention, also including executive functions, if different from those enhanced.

## Method

### Search Strategy

The review authors undertook a comprehensive search of databases as MEDLINE Advanced PsycINFO, EMBASE, CINHAL and CENTRAL (Cochrane Controlled Registered Trials) in April 2020, in accordance with the PRISMA statement (Moher et al., 2009). The search strategy comprised keywords in different combinations referring to four main clusters: “executive functions'', “neurodevelopmental disorders'', “children” “intervention” and “far effects'' including terms related to constructs and definitions (see Appendix 1 for complete search string and the Introduction for the definition of the specific terms). The keywords were selected based on the analysis of the literature on the effect of training in neurodevelopmental disorders (Kassai et al., 2019; Diamond e Ling in Novick et al., 2020; Scionti et al., 2020; Takacs & Kassai, 2019). The selection of terms referring to executive functions was guided by the models suggested by Diamond (2013) and Miyake et al. (2000). The latter one also includes emotional aspects such as emotion regulation and “hot” EF, which are considered also in this review as part of executive functions. Given the recent increased interest in studying the effects of EF training in children, the research was restricted to the period 2000–2020. In order to exclude non-peer reviewed studies, the authors included studies published in academic journals, reported in English and available for full text. The methodological quality of the included studies was assessed according to the National Health and Medical Research Council (NHMRC) Evidence Hierarchy (NHMRC, 2009).

### Inclusion Criteria

#### Type of Participants

Published studies included samples of subjects in developmental age (5–18 years) diagnosed with Neurodevelopmental Disorders (according to ICD 11 or 10 or DSM 5 or IV TR) They included Learning Disorders, Developmental Coordination Disorder, Language Disorder, Autism Spectrum Disorder, Attention Deficit Hyperactivity Disorder and Intellectual Disabilities (or as defined by the ICD or DSM IV). The choice of age range was guided by evidence described above that EFs develop from the first year of life to late adolescence, with a peak of development during the first 5 years of life (Garon et al., 2008). Furthermore, it is possible that for some neurodevelopmental disorders a clear diagnosis cannot be formulated before the age of five, thus, it is from the end of the preschool ages that eventual alterations in EFs are expected, and in turn, interventions are needed.

#### Type of Interventions

Selected studies focused on interventions aimed at improving any process belonging to the executive function domain (i.e., inhibition, working memory, shifting, planning, organization, problem solving, decision making, cognitive control, effortful control, self-regulation). Intervention could begin at any time during childhood, and it could have been carried out either in an ecological context, such as home or school, or in an experimental context, such as a laboratory. The intervention had to be carried out by health professionals (such as psychologists, neuropsychiatrists or occupational therapists) or by education professionals (such as teachers or educators). Types of interventions could include any program assumed to work on EFs, such as neurocognitive stimulation, neurocognitive training, computer programs, scholastic and academic curricula, occupational therapy, neuropsychological rehabilitation, psychoeducation, mindfulness and physical activities. Any frequency, intensity and duration of training was included. Moreover, the studies included needed to have a pre-post treatment design or the presence of a control group (active or waitlist).

#### Type of Outcomes

To be selected, studies must have measured far effect outcomes at the completion of the intervention.

The outcome variables had to be measured with standardized, objective tests administered to the child (either commercial or prototypal/experimental) and with parent’s and self-report questionnaires. These far effect measures included standardized neuropsychological and cognitive tests, achievement tests (math or reading or writing), quality of life questionnaires, self-regulation questionnaires, teachers’ ratings (school readiness, general literacy skills, or math or reading or writing), report cards (literacy or math or reading or writing).

Studies were excluded if: (1) they included single case studies and reviews; (2) they were diagnostic or prognostic studies (2) participants’ age was > 18 or < 5 or not clearly defined; (3) they included participants with other medical, psychiatric or neurological conditions not included in the classification of neurodevelopmental disorders, (4) the training was not targeted on cognitive or neuropsychological domains, (5) there was no control group, (6) there were no far effect outcome measures.

### Study Selection Process

**Fig. 1:**
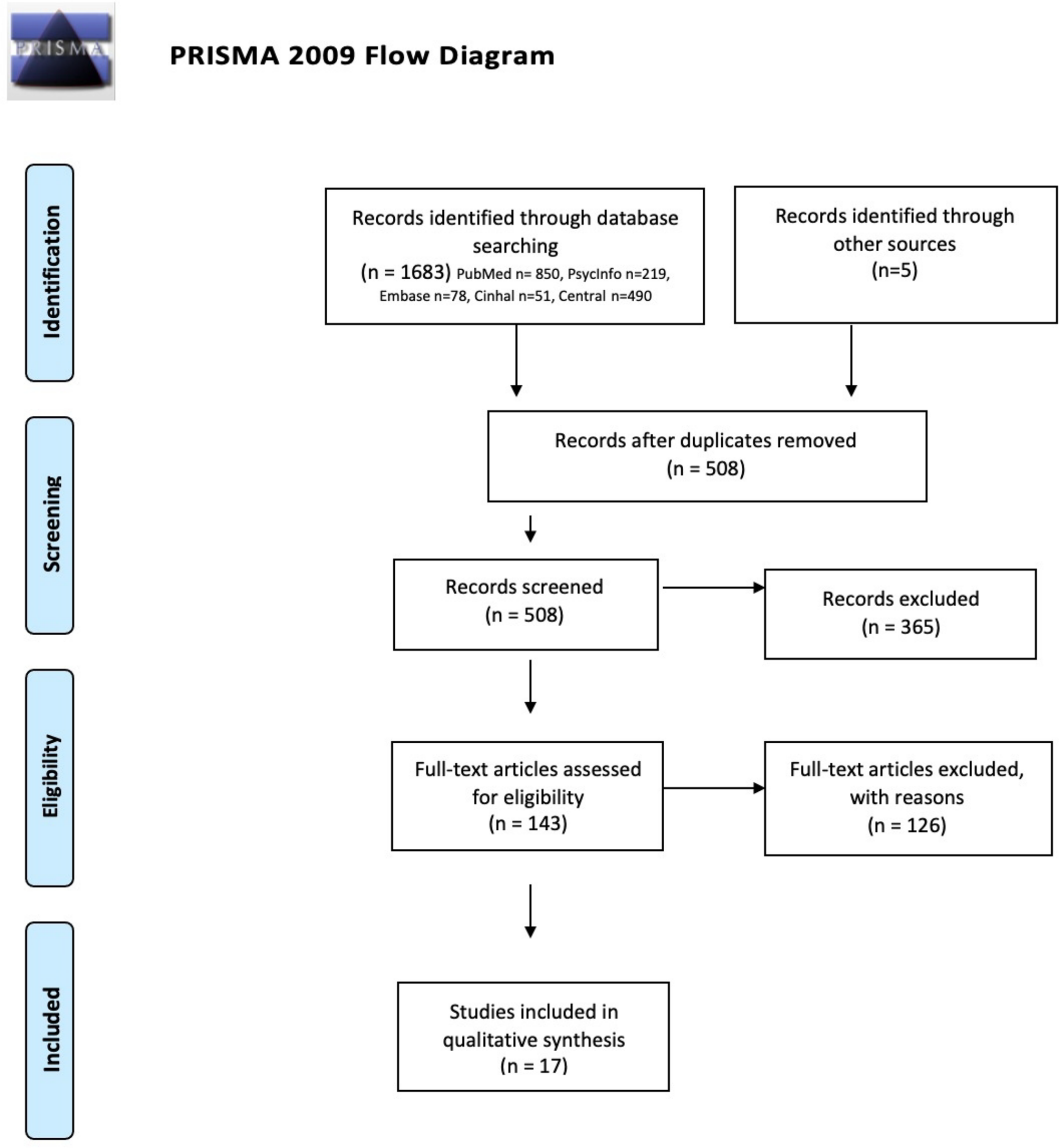
Study selection process following the PRISMA 2009 flow diagram

The initial literature searches produced 1683 papers. Five of these studies were included by analysing the articles’ bibliography. After removing duplicates, 508 articles were reviewed independently by three authors (Clara Bombonato (CB), Benedetta Del Lucchese (BDL), and Costanza Ruffini (CR)) on the basis of the title and abstract with an inter-rater agreement of 100%. 143 full-text articles were selected and reviewed to identify those that met the inclusion criteria. When discrepancy arose, articles were discussed and re-reviewed to determine their inclusion or exclusion. The process led to the selection of 17 papers that met the inclusion criteria. The overall process for selecting studies is shown in Fig. 1.

### Meta-analysis

Far effect outcome measures of reviewed studies including control groups were analyzed. The data collected from the articles were analyzed using software R, version 4.1.2. All of the studies included different outcomes, divided and analyzed on the basis of 5 macro categories considered as far effects, ;detailed in paragraph 3.5. A multivariate random-effect linear model, making use of Hedges Estimator, was used to conduct a meta-analysis. Hedge's g values were calculated and, ;according to Cohen (Cohen, 1977), values of effect sizes between 0.2 and 0.5 were considered "small", between 0.5 and 0.8 "medium", and > 0.8 "large". Effect size estimates were pooled across studies to obtain an overall effect size.

## Results

Seventeen studies were eligible for inclusion. The methodological quality of the included studies was independently assessed by the reviewers according to the National Health and Research Council (NHMRC). All studies were classified at level II, as Randomized Control Trials (Bigorra et al., 2016a, b; Bowling et al., 2017; Chacko et al., 2014; de Vries et al., 2015; Dovis et al., 2015; Egeland et al., 2013; Esmaili et al., 2019; Kenworthy et al., 2014; Kirk et al., 2016, 2017; Klingberg et al., 2005; Leins et al., 2007; Smith et al., 2020; Strehl et al., 2017; Weiss et al., 2018), except one that was classified at level III-1, as Pseudorandomized Control Trial (Beck et al., 2010).

### Participants

Studies including children with neurodevelopmental disorders as the target population of the intervention were selected. In particular, samples were composed by children with Attention Deficit and Hyperactivity (ADHD) in ten studies (Beck et al., 2010; Bigorra et al., 2016a, b; Chacko et al., 2014; Dovis et al., 2015; Egeland et al., 2013; Klingberg et al., 2005; Leins et al., 2007; Smith et al., 2020; Strehl et al., 2017), children with Autism Spectrum Disorder (ASD) in three studies, (de Vries et al., 2015; Kenworthy et al., 2014; Weiss et al., 2018), children with Intellectual and Developmental Disabilities (ID) in two studies (Kirk et al., 2016, 2017) and children with Specific Learning Disabilities in one study (SLD) (Esmaili et al., 2019). Moreover, it was agreed to include one study that targets children with Behavioral Health Disorders (BHD) (Bowling et al., 2017), since, although not present in the main diagnostic classifications (DSM-5; ICD-10), a broad category including some of the neurodevelopmental disorders mentioned above (ASD, ADHD). The studies also varied in terms of the age range of the population (4–17 years) and sample size (50 to 150 subjects).

### Study Design

Regarding the study design, in fifteen studies, the population was divided into two groups. In five of these studies, the control group underwent a training equivalent to that of the experimental group but non-adaptive, therefore without the adjustment for difficulty (Bigorra et al., 2016a, b; Chacko et al., 2014; Kirk et al., 2016, 2017; Klingberg et al., 2005), in four studies the control group consisted in the waitlist (Beck et al., 2010; Bowling et al., 2017; Esmaili et al., 2019; Weiss et al., 2018), in three studies the experimental group’s performance was compared with that of an active control group following an intervention not focused on EFs. Kenworthy et al., 2014; Leins et al., 2007; Strehl et al., 2017), and in two studies the control group received treatment as usual (Egeland et al., 2013; Smith et al., 2020). In two studies, the population was divided into three groups: two experimental groups and one control group, which underwent non-adaptive training (de Vries et al., 2015; Dovis et al., 2015).

### Intervention

All the selected articles provided results about an intervention aimed at executive functions rehabilitation. Such treatments were undertaken in several ways. Specifically, most of the interventions included computer training activities (Beck et al., 2010; Bigorra et al., 2016a, b; Chacko et al., 2014; de Vries et al., 2015; Dovis et al., 2015; Egeland et al., 2013; Kirk et al., 2016, 2017; Klingberg et al., 2005); in addition, among the selected articles there were two neurofeedback treatments (Leins et al., 2007; Strehl et al., 2017), two curriculum interventions delivered during school attendance (Kenworthy et al., 2014; Smith et al., 2020), an individualized manualized Cognitive Behavioural Therapy (CBT) intervention (Weiss et al., 2018), a training delivered through cooperative and collaborative group play activities at the clinic (Esmaili et al., 2019) and finally an intervention based on physical activity (Bowling et al., 2017).

In most studies, the intervention targeted cold components of executive functions, specifically working memory (Beck et al., 2010; Bigorra et al., 2016a, b; Chacko et al., 2014; de Vries et al., 2015; Dovis et al., 2015; Egeland et al., 2013; Esmaili et al., 2019; Klingberg et al., 2005; Smith et al., 2020), inhibition (Dovis et al., 2015; Esmaili et al., 2019; Leins et al., 2007; Smith et al., 2020) and attentional control Kirk et al., 2016, 2017; Leins et al., 2007; Smith et al., 2020), while others aimed at strengthening other executive functions such as planning, problem-solving, shifting, monitoring and cognitive flexibility (de Vries et al., 2015; Dovis et al., 2015; Esmaili et al., 2019; Kenworthy et al., 2014). Four studies targeted the hot components of executive functions, in particular self-regulation and emotional regulation, as intended by the Miyake et al. model (2000) (Bowling et al., 2017; Esmaili et al., 2019; Strehl et al., 2017; Weiss et al., 2018).

These interventions were carried out in different settings; at home in ten studies (Beck et al., 2010; Bigorra et al., 2016a, b; Chacko et al., 2014; de Vries et al., 2015; Dovis et al., 2015; Kirk et al., 2016, 2017; Klingberg et al., 2005; Weiss et al., 2018), at school in five studies (Bowling et al., 2017; Egeland et al., 2013; Kenworthy et al., 2014; Smith et al., 2020; Weiss et al., 2018) and at the clinic in three studies (Esmaili et al., 2019; Leins et al., 2007; Strehl et al., 2017).

The duration of the interventions ranged from 5 weeks to 3 months; only one study involved a treatment in which the 28 sessions were spread over a year (Kenworthy et al., 2014).

The intensity of the intervention varied from 2 times a week to daily, twice a week in three studies (Bowling et al., 2017; Esmaili et al., 2019; Strehl et al., 2017), 3–4 times a week in one study (. Smith et al., 2020), 5 times a week in seven studies (Bigorra et al., 2016a; Chacko et al., 2014; Kirk et al., 2016, 2017; Klingberg et al., 2005; Leins et al., 2007). In one study, frequency of intervention corresponded to the total days of school attendance (Egeland et al., 2013). Of the articles examined, five studies did not report the frequency of intervention but the overall duration of the intervention: 6 weeks (de Vries et al., 2015), 10–14 weeks (Weiss et al., 2018), 28 sessions (Kenworthy et al., 2014), 25 sessions over 5–6 weeks (Beck et al., 2010) and the last one, 25 sessions over 5 weeks (Dovis et al., 2015). The duration of each single treatment session ranged from 20 min to 2 h.

### Far Effect Outcomes

According to the research questions of the studies, different far effects were measured. However, it was possible to outline some common aspects that had been investigated, regardless of the type and target of the author's intervention. Most of the authors investigated whether, as a consequence of training on specific executive functions, improvements were obtained on other executive functions not directly trained. For example, Bigorra and colleagues (2016a, b) conducted two interventions on working memory and explored the far effect on inhibition, sustained attention, planning, cognitive flexibility, task switching (study 1) and decision making (study 2). De Vries and colleagues (2015) explored inhibition, sustained attention, working memory or cognitive flexibility and their intervention was directed to working memory or cognitive flexibility. Dovis and colleagues (2015) led a training on visuospatial working memory, inhibition and cognitive flexibility and studied the far effect on interference control, verbal short-term memory/working memory and complex reasoning. For Egeland and colleagues (2013) working memory was the target intervention and processing speed, attention, inhibitory control were assessed as far effects. Klingberg and colleagues (2005) implemented a working memory training and studied inhibition as a far effect. All these studies implemented neuropsychological outcome measures. Finally, Kirk and colleagues’ (2017) intervention target was attentional control and response inhibition while Beck and colleagues’ (2010) was working memory and both studies investigated parent and teacher-report child daily executive functioning as outcome measures.

Another common target of investigation was the study of any changes, following the training, in the disorder’s specific symptomatology: ADHD symptoms (Beck et al., 2010; Bigorra et al. 2016a, b; Chacko et al., 2014; de Vries et al., 2015; Dovis et al., 2015; Egeland et al., 2013; Kirk et al., 2016; Klingberg et al., 2005; Leins et al., 2007; Smith et al., 2020; Strehl et al., 2017), autism symptoms ( Kenworthy et al., 2014), mood (Weiss et al., 2018) referred by parents and teachers or by the clinician (Smith et al., 2020; Strehl et al., 2017; Weiss et al., 2018).

The majority of studies assessed the child’s daily life functioning, including adaptive behaviour (Bigorra et al., 2016a, b; Dovis et al., 2015; Egeland et al., 2013; Kirk et al., 2017; Leins et al., 2007; Strehl et al., 2017; Weiss et al., 2018), quality of life (de Vries et al., 2015; Dovis et al., 2015; Esmaili et al., 2019; Strehl et al., 2017), classroom functioning (Bowling et al., 2017; Kenworthy et al., 2014), and social skills (Bigorra et al., 2016a, b; de Vries et al., 2015).

A recurring aspect that was investigated with direct outcome measures was the child’s learning skills such as reading comprehension (Bigorra et al., 2016a, b), math, vocabulary, letter knowledge and rhyme detection (Kirk et al., 2017), reading and math (Egeland et al., 2013), word reading, sentence comprehension, spelling, and mathematical computation (Chacko et al., 2014).

Finally, a few studies explored other cognitive outcomes as far effect: memory (Egeland et al., 2013), complex non-verbal reasoning (Dovis et al., 2015; Klingberg et al., 2005; Strehl et al., 2017) and intelligence (Leins et al., 2007).

### Efficacy on Far Effects

Results will be presented dividing the selected articles according to the EF component target of the intervention. Within each section, the studies will be reported analyzing the far effects investigated, which are categorized into 5 macro categories, agreed upon by the authors of this systematic review. These macro-categories grouped the different outcomes assessed as far effects (other executive functions, clinical symptoms, learning skills, daily life functioning and cognitive outcomes).

#### Intervention on Attentional Control and Inhibition

KIRK et al. (2016), Kirk et al. (2017), Leins et al. (2007) analysed the effects of interventions targeting attention and inhibition (Table 1).

**Table 1:** Studies implementing interventions on attentional control and inhibition

**Authors**	**Study design**	**Diagnosis**	**Population**	**Age**	**Intervention**	**Target of intervention**	**Duration**	**Intensity**	**Assessment of near effect**	**Assessment of far effect**	**Near effects**	**Far effects**
Kirk et al. (2017)	RCT	ID	EG n = 38, CG n = 37 (non-adaptive training)	4–11 yrs	Training Attention and Learning Initiative (TALI): computer training at home	Selective attention, sustained attention, attentional control (conflict resolution; response inhibition)	5 wks (25 sessions)	20 min per day, 5 times a week	Not investigated	Learning skills: GAN, TEMA-3, PPVT-4, PAT (letter knowledge and rhyme detection subscales) Other executive Functions: BRIEF, WMRS Daily life functioning: DBC-P	-	Yes, on mathematical learning skills (TEMA) at 3 months follow-up No other learning skills, executive functions non-trained and daily life functioning
Kirk et al. (2016)	RCT	ID	EG n = 38, CG n = 37 (non-adaptive training)	4–11 yrs	Training Attention and Learning Initiative (TALI): computer training at home	Selective attention, Sustained attention, attentional control (conflict resolution; response inhibition)	5 wks (25 sessions)	20 min per day, 5 times a week,	Selective attention, attentional control and sustained attention: WATT (visual search task; sustained attention task)	Clinical Symptoms: SWAN	Yes, on selective attention (Number of errors) No on attentional control, selective attention (time) and sustained attention	No significant treatment effect found
Leins et al. (2007)	RCT	ADHD	EG1 (SCP) n = 19, EG2 (Theta/beta) n = 19	8–13 yrs	Neurofeedback in clinic	Attention, inhibition	2 wks (10 sessions) for three treatment phases with a break of 4 to 6 weeks between each phase	1 h per session	Attention: TAP	Clinical symptoms: DSM-IV—questionnaires for parents and teachers, Conners’ Rating Scale Daily life functioning: ECBI Cognitive outcomes: HAWIK-III;	Yes, on attention for both EGs	No differences between EG on clinical symptoms (Conners’ Rating Scale, DSM IV questionnaires), on daily life functioning (ECBI) and on Cognitive outcomes (HAWIK-III). At post hoc analysis significant improvement only for EG 2 on cognitive outcomes and on clinical symptoms

Legend: *RCT* Randomized Controlled Trial, *ID* Intellectual Disability, *ADHD* Attention Deficit Hyperactivity Disorder, *EG* Experimental Group, *CG* Control Gropu, *DSM IV* Diagnostic and Statistical Manual of mental disorders, *WATT* Wilding attention battery, *TAP* Testbatterie zur Aufmerksamkeitsprufung, *GAN* Give A Number, *TEMA* Test of Early Mathematics Ability, *PPVT-4* Peabody Picture Vocabulary Task-4, *PAT* Phonological Abilities Test, *BRIEF* Behavior Rating Inventory of Executive Functions, *WMRS* Working Memory Rating Scale, *DBC-P* Developmental Behavior Checklist Parent, *SWAN* Strengths and Weaknesses of ADHD symptoms and Normal behavior scale, *ECBI* Eyberg Child Behavior Inventory, *HAWIK III* The Hamburg-Wechsler intelligenztest fur Kinder

One study ( Kirk et al., 2017) evaluated improvement of executive functions in daily life with parent and teacher report questionnaires, finding no significant far effect of the computerized attentional training on children with intellectual disability.

Two studies evaluated a reduction of ADHD symptomatology (rating scales) as a far effect of the interventions. Kirk et al. (2016) found no significant effects of the computerized attentional training in children with intellectual and developmental disabilities, while Leins et al. (2007), in children with ADHD found a significant reduction in symptoms after neurofeedback interventions in the two experimental groups, but in absence of a control group and without observing specific differences between the two types of treatment.

Only Kirk et al. (2017) considered the improvement in learning skills (defined as both academic skills and as abilities supporting learning) as a far effect, finding significant effects only for mathematic skills at the three-month follow-up, while no effects were found in cognitive skills underlying school learning, such as the receptive vocabulary and metaphonological skills neither at the post-test nor at the follow-up assessment.

The two studies, which evaluated children’s daily life functioning through parent report questionnaires, did not find significant effects, neither in terms of improvement of behavioural and emotional problems ( Kirk et al., 2017), nor of behavioural problems at home (Leins et al., 2007).

Leins et al. (2007) evaluated cognitive functioning (German intelligence test for children) as a far effect of the intervention, finding a significant increase in both neurofeedback intervention groups; however, these results were not compared with any control group.

#### Intervention on Working Memory

Seven of the studies (Beck et al., 2010; Bigorra et al., 2016a, b; Chacko et al., 2014; de Vries et al., 2015; Egeland et al., 2013; Klingberg et al., 2005) analysed the effects of interventions targeting working memory (Table 2).

**Table 2:** Studies implementing interventions on working memory

**Authors**	**Study design**	**Diagnosis**	**Population**	**Age**	**Intervention**	**Target of intervention**	**Duration**	**Intensity**	**Assessment of near effect**	**Assessment of far effect**	**Near effects**	**Far effects**
Bigorra et al. (2016a)	RCT	combined-type ADHD	EG n = 36, CG (non-adaptive CogMed training) n = 30	7–12 yrs	CogMed computer training at home	Spatial and verbal WM	5 wks	5 sessions for week	WM: WISC IV (Digit Span Backward, letter-Number Sequencing), WMS-III (Spatial span backward) BRIEF (WM subscale)	Other Executive functions: BRIEF, CPT II, ToL, WCST-64 and TMT B Clinical symptoms: Conners' rating scales-revised, CBCL/4–18, TRF/4–18 Daily life functioning: SDQ, WFIRS-P Learning skills: Canals	Yes, on BRIEF WM subscale and on a WM composite score	Yes, on other executive functions (BRIEF, CPT), clinical symptoms (composite score) and daily life functioning (school learning behavior at WFIRS-P, only at follow-up) No on learning skills
Bigorra et al. (2016b)	RCT	combined-type ADHD	EG n = 36, CG (non-adaptive CogMed training) n = 30	7–12 yrs	CogMed computer training at home	Spatial and verbal WM	5 wks	5 sessions for weeks	WM: WISC IV (Digit Span Backward, Letter-Number Sequencing), WMS-III (Spatial span backward)	Daily life functioning: Happè’s Strange Stories, Folk Psychology Test Other Executive function: IGT	Yes, the results are reported in the previous article (Bigorra et al., 2016a, 2016b)	No significant treatment effects found
De Vries et al. (2015)	RCT	ASD	EG1 (WMtr) n = 40, EG2 (FLEXtr) n = 37, CG (non-adaptive mock training) n = 38	8–12 yrs	Braingame Brian: computer training at home	EG1: 5 WM activities with increasing difficulties (remembering, manipulating and updating). EG2: One Cognitive Flexibility activity with increasing difficulty	6 wks (25 sessions)	Not reported	WM: Corsi-BTT (similar to activities' training), N back task (different to activities' training); Cognitive Flexibility: GEWT (similar to activities' training), NGST (different to activities' training)	Other executive functions: Stop task, SART, BRIEF Daily Life Functioning: CSBQ, PedsQL Clinical symptoms: DBDRS	No significant differences between groups for working memory and cognitive flexibility	No significant treatment effects found
Egeland et al. (2013)	RCT	ADHD	EG (TAU + Cogmed) n = 38, CG (TAU) n = 37	10–12 yrs	CogMed computer training at school	Spatial and verbal WM	5–7 wks	Each school day (30–45 min)	WM: BVRT	Other executive functions: CW, TMT (D-KEFS), BRIEF, CCPT-II Cognitive outcomes: CAVLT-2 Learning skills: Key Math, LOGOS Clinical symptoms: ARS-IV Daily life functioning: SDQ	No	Yes, on reading learning skills No on cognitive outcomes (CAVLT-2), maths learning skills, other executive functions (CW, TMT (D-KEFS), BRIEF, CCPT-II), clinical symptoms (ARS-IV) and daily life functioning (SDQ)
Chacko et al. (2014)	RCT	ADHD	EG n = 44, CG (Non adaptive training) n = 41	7–11 yrs	Cogmed computer training at home	verbal and non-verbal WM	25 sessions	Fine days per week (30–45 min)	WM: AWMA	Clinical symptoms: DBD, actigraphs Learning skills: WRAT4-PMV	Yes, on WM (non-verbal and verbal storage) but no significant differences between groups on measures of nonverbal or verbal complex working memory (storage plus processing/manipulation)	No significant treatment effects found
Klingberg et al. (2005)	RCT	ADHD	EG n = 26, CG (non adaptive WM training) n = 24	7–12 yrs	Cogmed computer training at home	Spatial and verbal WM	5–6 wks (25 sessions)	40 min per day every day	Visuo-spatial WM: span-board task Verbal WM: Digit-span (WISC III)	Clinical symptoms: parent and teacher-report Conners Rating Scale, number of head movements Cognitive outcomes: CPM Other executive functions: Stroop Task	Yes, on visuo-spatial and verbal WM	Yes, on other executive functions (Stroop task), cognitive outcomes (CPM) and on parent ratings clinical symptoms No on teacher rating clinical symptoms
Beck et al. (2010)	NRS	combined type or inattentive type ADHD	EG n = 27, CG (waitlist) n = 25	7–17 yrs	Working Memory Training Program: computer training at home	Spatial and verbal WM	5–6 wks (25 sessions)	30–40 min per session	WM: BRIEF (WM subscale)	Other executive functions: BRIEF (other subscales) Clinical symptoms: P-ChIPS, Conners’ Rating Scale—teacher and parents	Yes, on WM (for parents at post training and at 4-month follow up, for teachers only at follow up)	Yes, on clinical symptoms (ChIPS, Conners’ Rating Scale) and on other executive functions (BRIEF)

Legend: *RCT* Randomized Controlled Trial, *ADHD* Attention Deficit Hyperactivity Disorder, *ASD* Autism Spectrum Disorder, *EG* Experimental Group, *CG* Control Gropu, *DSM IV* Diagnostic and Statistical Manual of mental disorders, *WM* Working Memory, *WMtr* Working memory training, *TAU* Treatment As Usual, *WISC IV* Wechsler Intelligence Scale for Children-IV, *WMS III* Wechsler Memory Scale-III, *BRIEF* Behavior Rating Inventory of Executive Functions, *Corsi – BTT* Corsi Block Tapping Task, *GEWT* Gender Emotion Switch Task, *NGST* Number gnome switch task, *BVRT* Benton Visual Retention Test, *AWMA* The Automatic Working Memory Assessment, *CCPT-II* Conners’ Continuous Performance Test-II, *TOL* Tower of London, *WSCT* Wisconsin Card Sorting Test, *TMT – B* Trail Making Test, *CBCL* Child Behaviour Checklist, *TRF* Teacher’s report Form/4-18, *SDQ* Strenght and Difficulties Questionnaire, *WFIRS* Weiss Functional Impairment rating scale for parents, *IGT* Iowa Gambling Task, *SART* Sustained attention response task, *CSBQ* Children's Social Behavior Questionnaire, *PedsQL* The Pediatric Quality of Life Inventory, *DBDRS* parent version of the Disruptive Behavior Disorders Rating Scale, *CW* Color Word, *DKEFS* Delis-Kaplan Executive Function System, *CAVLT-2* Children’s Auditory Verbal Learning Tests-2, *ARS ADHD* Rating Scale, *WRAT4-PMV* Wide Range Achievement Test 4 Progress Monitoring Version, *CPM* Colour Progressive Matrices, *ChIPS* Children’s Interview for Psychiatric Syndromes–Parent Form

Among the six studies that included other executive functions, assessed with neuropsychological measures, as far effects of the intervention in children with ADHD, three found significant effects on response inhibition (Bigorra et al., 2016a, b; Egeland et al., 2013; Klingberg et al., 2005), one on sustained attention (Bigorra et al., 2016a, b), and one on cognitive flexibility (Egeland et al., 2013). On the contrary, Bigorra et al. (2016b) found no significant effects in improving decision making and De Vries et al. (2015) found no significant effects on sustained attention, inhibition, and cognitive flexibility in children with ASD. Some studies assessed far effects on other executive functions by means of parent or teacher report questionnaires (BRIEF), finding significant effects (Beck et al., 2010; Bigorra et al., 2016a, b) indicating an improvement on executive functions in ecological settings. On the other hand, Egeland et al. (2013) and De Vries et al. (2015) reported no significant effect of the intervention in increasing executive functioning in daily life.

Among the six studies that included the reduction of clinical symptoms, measured with teacher or parent-report, as a far effect of working memory interventions, three studies found a significant reduction in ADHD-related symptoms in children with this neurodevelopmental disorder (Beck et al., 2010; Bigorra et al., 2016a, b; Klingberg et al., 2005). In contrast, other studies did not find significant effects in reducing ADHD-clinical symptoms neither in children with ADHD (Chacko et al., 2014; Egeland et al., 2013) nor in children with ASD (de Vries et al., 2015). Furthermore, when ADHD symptomatology was assessed with direct measures, as attention, activity level and impulse control measured by actigraphs (Chacko et al., 2014) and by the number of head movements measured by an infrared camera (Klingberg et al., 2005), no significant far effects were reported.

Among the three studies that evaluated the improvement of learning skills as a far effect of the intervention, only Egeland et al. (2013) found significant effects in improving speed and accuracy of reading. No significant effects were found in improving reading comprehension (Bigorra et al., 2016a, b), math skills (Egeland et al., 2013), word reading, sentence comprehension, spelling, and mathematical computation (Chacko et al., 2014).

Among the four studies that evaluated functioning in daily life (behaviour, social skills, quality of life), only Bigorra et al., (2016a, b) found a significant effect in improving school learning behaviour (i.e. need for an extra help at school, grades that are below potential), assessed through a parent report questionnaire, while no significant effects were found in improving behavioural and emotional skills (Bigorra et al., 2016a, b; Egeland et al., 2013), social skills (de Vries et al., 2015) or quality of life (de Vries et al., 2015). Finally, a direct test assessing of theory of mind skills (Bigorra et al., 2016a, b) did not yield any improvement.

In the two studies that considered an improvement in cognitive processes as a far effect of the intervention, Klingberg et al. (2005) found a significant effect in improving non-verbal reasoning abilities (Raven’s Matrices), while Egeland et al. (2013) found no significant effects on auditory long-term memory (word recall and recognition).

#### Intervention on Cognitive Flexibility

Only one of the studies included in this systematic review analysed the effects of a treatment aimed at improving cognitive flexibility (de Vries et al., 2015) (Table 3). No significant far effects were reported for children with ASD: neither on other executive functions assessed through questionnaires and standardized tests, nor on clinical symptoms, daily life functioning, or on quality of life.

**Table 3:** Studies implementing intervention on cognitive flexibility

**Authors**	**Study design**	**Diagnosis**	**Population**	**Age**	**Intervention**	**Target of intervention**	**Duration**	**Intensity**	**Assessment of near effect**	**Assessment of far effect**	**Near effects**	**Far effects**
De Vries et al. (2015)	RCT	ASD	EG1 (WMtr) n = 40, EG2 (FLEXtr) n = 37, CG (non-adaptive mock training) n = 38	8–12 yrs	Braingame Brian: computer training at home	EG1: 5 WM activities with increasing difficulties (remembering, manipulating and updating). EG2: One Cognitive Flexibility activity with increasing difficulty	6 wks (25 sessions)	Not reported	WM: Corsi-BTT (similar to activities' training), N back task (different to activities' training); Cognitive Flexibility: GEWT (similar to activities' training), NGST (different to activities' training)	Other executive functions: Stop task, SART, BRIEF Daily Life Functioning: CSBQ, PedsQL Clinical symptoms: DBDRS	No significant differences between groups for working memory and cognitive flexibility	No significant treatment effects found

Legend: *RCT* Randomized Controlled Trial, *ASD* Autism Spectrum Disorder, *EG* Experimental Group, *CG* Control Group, DSM IV, *WM* Working Memory, *WMtr* Working memory training, *FLEXtr* Flexibility training; Corsi – BTT Corsi Block Tapping Task, *GEWT* Gender Emotion Switch Task, *NGST* Number gnome switch task, *SART* Sustained attention response task, *BRIEF* Behavior Rating Inventory of Executive Functions – parent, *CSBQ* Children's Social Behavior Questionnaire, *PedsQL* The Pediatric Quality of Life Inventory, *DBDRS* parent version of the Disruptive Behavior Disorders Rating Scale

#### Intervention on Hot Executive Functions

Three studies (Bowling et al., 2017; Strehl et al., 2017; Weiss et al., 2018) investigated the effects of interventions (physical activity through virtual reality, emotional regulations trainings, neurofeedback) aimed at improving the "hot" component of executive functions on clinical symptomatology, daily life functioning and intelligence in children with different neurodevelopmental disorders (Table 4).

**Table 4:** Studies implementing interventions on hot executive functions

**Authors**	**Study design**	**Diagnosis**	**Population**	**Age**	**Intervention**	**Target of intervention**	**Duration**	**Intensity**	**Assessment of near effect**	**Assessment of far effect**	**Near effects**	**Far effects**
Bowling et al. (2017)	RCT	BHD (ASD, ADHD, anxiety disorders, depressive disorders)	EG n = 52, CG (waitlist) n = 52	7–16 yrs	Manville Moves: VR-cybercycling at school	Self-regulation	7 wks	2 sessions per week (30–40 min)	Behavioural self-regulation: CATRS-10	Daily life functioning: classroom functioning (TOC per days)	Yes	Yes
Weiss et al. (2018)	RCT	ASD	EG n = 31, CG (waitlist) n = 29	8–12 yrs	Secret Agent Society: Operation Regulation at home and school	Emotional regulation	10–14 wks	Not reported	Emotional regulation: ERSSQ-P, ERC, CEM, Dylan, James	Daily life functioning: BASC-2 Clinical symptoms: ADIS-P, CGI-S, BASC-2	Yes, on parent report emotional regulation measures No significant group differences on any of the child-reported ER measures	Yes, on daily life functioning (BASC -2) and clinical symptoms (ADIS-P, CGI-S) Gains maintained at follow-up
Strehl et al. (2017)	RCT	ADHD	EG1 (Neurofeedback) n = 76, EG2 (EMG Feedback) n = 74	7–9 yrs	Neurofeedback; EMG feedback in clinics	Self-regulation	3 months (25 sessions with a break after 12 sessions of 4–6 weeks.)	2–3 sessions per week	Cortical self-regulation ADHD symptoms: German ADHD rating scale (subscale inattention, hyperactivity and impulsivity)	Clinical symptoms: Parents’ ratings of ADHD subdomains, Teachers’ ratings of ADHD symptoms,CGI-I Daily life functioning: SDQ, Kid-KINDL Cognitive outcomes: CPM	Yes, EG1 was significantly superior to EMG in reducing ADHD core symptoms	Yes, there are significant differences between EG groups on clinical symptoms (Parents’ Ratings of ADHD Subdomains on impulsivity and inattention) and on cognitive outcomes (CPM) No, there are not differences between EG groups on clinical symptoms (Teachers’ Ratings of ADHD Core Symptoms and CGI-I) and on daily life functioning (Kid-KINDL,SDQ)

Legend: *RCT* Randomized Controlled Trial, *ASD* Autism Spectrum Disorder, *ADHD* Attention Deficit Hyperactivity Disorder, *BHD* behavioral health disorders, *EG* Experimental Group, *CG* Control Group, *EMG* Electromiography, *VR* Virtual Reality, *CATRS* Conner's abbreviated teacher rating scale, *ERSSQ-P* Emotion Regulation and Social Skills Questionnaire, *ERC* Emotion Regulation Checklist, *CEM* Children’s Emotion Management Scales, *TOC* Time out of class, *BASC-2* Behavior Assessment System for Children, Second Edition – Parent Rating Scales, *ADIS-P* Anxiety Disorders Interview Schedule – Parent Version, *CGI* Clinical Global Impression, *SDQ* Strenght and Difficulties Questionnaire, *Kid-KINDL* German quality of life assessment for kids, *CPM* Colour progressive matrices

Two studies evaluated the improvement of clinical symptoms as a far effect of the intervention. Specifically, Weiss et al., (2018) found significant effects in the improvement of symptomatology related to mood and behavioural disorders through parent report questionnaires and in the global clinical assessment evaluated by clinicians, while Strehl et al. (2017) found significant effects in terms of a decrease of inattention and hyperactivity from the analysis of teacher and parent report questionnaires, while there was no significant effect as expressed by the clinicians Global Clinical Impression (CGI).

All three studies evaluated functioning in daily life as a far effect of the intervention, finding significant effects on classroom functioning (Bowling et al., 2017) and on emotional and behavioural problems perceived by parents (Weiss et al., 2018). Instead, the neurofeedback intervention (Strehl et al., 2017) yielded no significant effects on the reduction of behavioural and emotional impairments assessed by parents and teachers or on the quality of life.

Only Strehl et al. (2017) evaluated cognitive outcomes, finding significant effects in improving non-verbal reasoning (Raven’s Matrices) in the neurofeedback group compared to the electromiography feedback group.

#### Integrated Intervention on Different EF Components

Four (Dovis et al., 2015; Esmaili et al., 2019; Kenworthy et al., 2014; Smith et al., 2020) of the studies investigated the effects of integrated trainings, that is, interventions simultaneously training different components of executive functions in children with different neurodevelopmental disorders (Table 5).

**Table 5:** Studies implementing integrated interventions

**Author**	**Study design**	**Diagnosis**	**Population**	**Age**	**Intervention**	**Target of intervention**	**Duration**	**Intensity**	**Assessment of near effect**	**Assessment of far effect**	**Near effects**	**Far effects**
Esmaili et al. (2019)	RTC	SLD	EG n = 28, CG (waitlist) n = 28	7–11 yrs	Peer- activities in groups: cooperative and collaborative plays in clinic	Inhibition, shifting, emotional control, working memory, initiation, planning, organization of materials, and monitoring	9 wks	2 sessions per week (3 h)	Executive functions: BRIEF	Daily life functioning: COSA	Yes	No significant treatment effects found
Kenworthy et al. (2014)	RCT	ASD	EG n = 47, CG (SS intervention) n = 20	7–11 yrs	Unstuck and On Target (UOT): curriculum at school	Flexibility, goal-setting, planning, using internalized language to support problem-solving	1 year (28 sessions for children, 1 session for parents and 1 session for teachers)	30–40 min	Problem solving: BD Flexibility and planning: CT Executive Functions: BRIEF	Clinical symptoms: SRS Daily life functioning: Classroom functioning (Classroom Observations Coding Form)		Yes on daily life functioning (classroom functioning) No on clinical symptoms
Smith et al. (2020)	RCT	ADHD	EG n = 48, CG (TAU) n = 44	5–9 yrs	Integrated Brain, Body and Social intervention (IBBS) at school	EF: Sustained attention, response inhibition, working memory, directed attention, attentional switching, divided attention, visual searching, OTHER: category formation, speed of processing, oppositional behavior, disruptive behavior	15 wks (60 sessions)	In USA: 3–4 days per week (2 h); In China: 3 days per week (90 min)	Memory and Learning: CVLT, WRAML-2 Interference control: Flanker task	Clinical symptoms: CGI-I, SNAP	Yes on memory and learning (CVLT) No significant treatment effect on memory and learning (WRAML-2) and interference control	No significant treatment effects found
Dovis et al. (2015)	RCT	combined-type ADHD	EG1 (full active condition) n = 31, EG2 (partially active condition) n = 28, CG (placebo non adaptive condition) n = 30	8–12 yrs	Braingame Brian: computer training at home	EG1: visuospatial WM, Inhibition and cognitive flexibility EG2: inhibition and cognitive flexibility	5 wks (25 sessions)	Not reported (35–50 min per session)	Visuospatial short-term memory and WM: CBTT Inhibition: Stop Task Cognitive Flexibility: TMT	Interference control: Stroop Color and Word Test Other executive functions: Digit span, BRIEF Cognitive outcomes: CPM Clinical symptoms: DBDRS Daily life functioning: PedsQL, SPSRQ-C, HSQ	Yes: EG1 improved on visuospatial short-term memory, WM and inhibition; EG2 improved on inhibition, but not on visuospatial short-term memory and WM	Yes there are significant differences between EG groups on other executive functions (interference control) No, there are not significant differences between EG groups on other executive functions (Digit Span, BRIEF), cognitive outcomes (CPM), clinical symptoms (DBDRS) and daily life functioning (PedsQL, SPSRQ-C, HSQ)

Legend: *RCT* Randomized Controlled Trial, *ASD* Autism Spectrum Disorder, *ADHD* Attention Deficit Hyperactivity Disorder, *SLD* Specific Learning Disability, *EG* Experimental Group, *CG* Control Group, *TAU* Treatment As Usual, *SS* Social Skills intervention, *WM* Working Memory, *BRIEF* Behavior Rating Inventory of Executive Functions, *BD* Block design, *CT* Challenge Task, *CVLT* Verbal Learning and Memory, *WRAML-2* Wide Range Assessment of Memory and Learning–Second Edition—visuo spatial memory feed foward and backward, *CBTT* Corsi Block Tapping Task, *TMT* Trail Making Test, *COSA* Child Occupational Self-Assessment, *SRS* Social Responsiveness Scale, *CGI-I* The Clinical Global Impression-Improvement, *SNAP* The Swanson, Nolan and Pelham Teacher and Parent Rating Scale, *CPM* Colour Progressive Matrices, *DBDRS* Disruptive Behavior Disorders Rating Scale, *PedsQL* Pediatric Quality of Life Inventory, *SPSRQ-C* Sensitivity to Punishment and Sensitivity to Reward Questionnaire for children, *HSQ* Home Situations Questionnaire

Dovis et al. (2015) evaluated the improvement in other executive functions than the target ones, in children with ADHD finding no significant effects either in the improvement of verbal working memory evaluated through standardized direct tests, or in executive functioning in the context of daily life evaluated through parent report questionnaires.

Among the three studies that evaluated the reduction of clinical symptoms as a far effect of the intervention, Dovis et al. (2015) found significant effects in ADHD behaviour perceived by teachers, but not by parents, while Smith et al. (2020) found no significant reduction in ADHD symptoms as assessed by clinicians, nor as perceived by parents and teachers in children with ADHD. Finally, Kenworthy et al. (2014) found no significant reduction in ASD symptoms in children with this disorder.

Kenworthy et al. (2014) found ASD after the intervention, assessed by an external blind researcher using observational measures. Instead, Esmaili et al. (2019) in children with specific learning disability, found no significant effects in children’s perceived competence in everyday activities, and Dovis et al. (2015) found no significant effects, in children with ADHD in improving children's motivational behaviours, neither in decreasing problematic behaviours at home and in public situations as assessed by parent report questionnaires nor in quality of life.

Only Dovis et al. (2015) evaluated the improvement of cognitive abilities, finding no significant effects in the improvement of non-verbal reasoning skills (Raven’s Matrices).

### Metanalysis Results

#### Non-trained Executive Functions

**Fig. 2:**
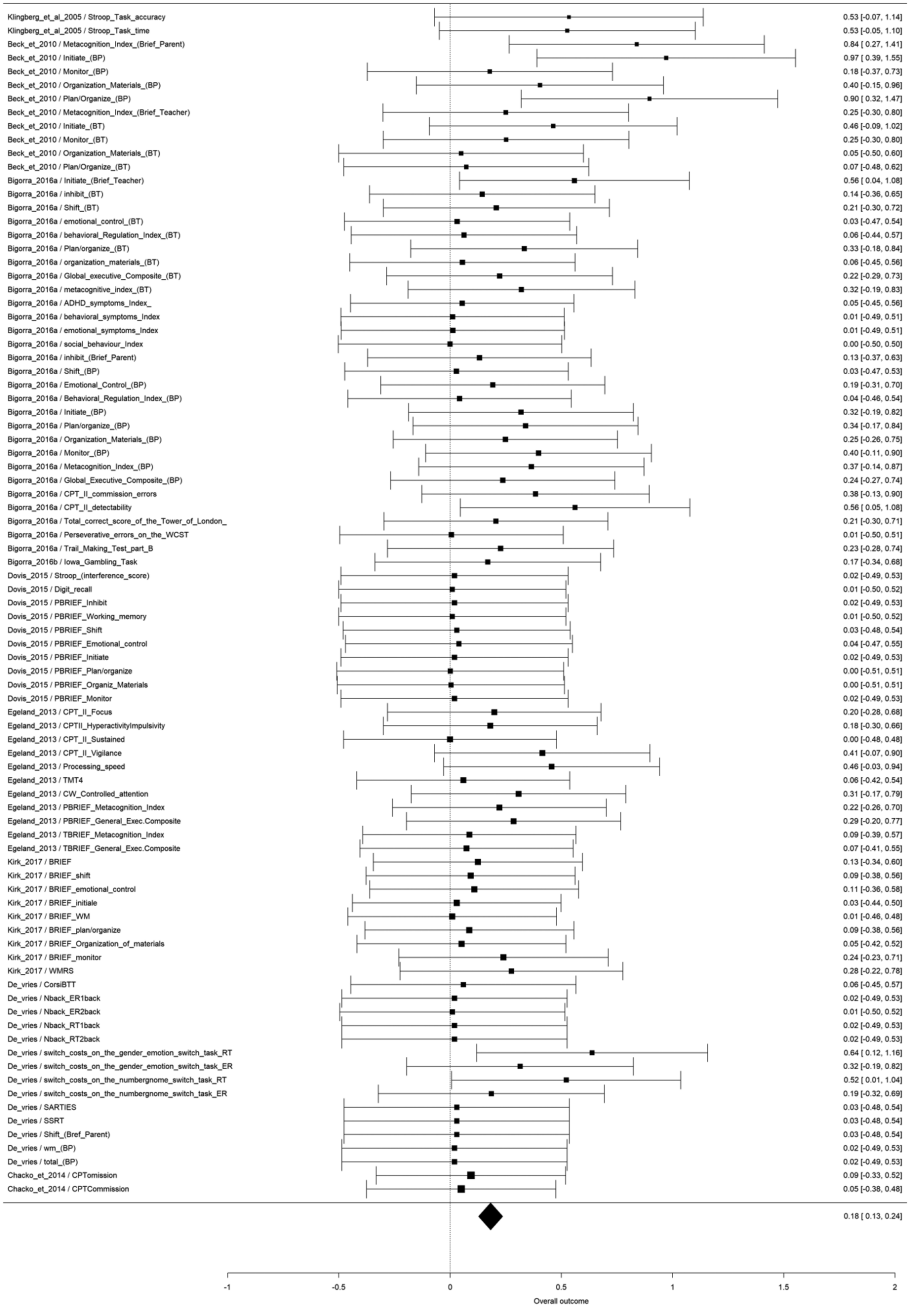
Metanalysis results of far effect on other executive functions

All of the 9 studies that assessed a non-trained EF as far effect was included in the metanalysis, considering 87 outcome measures. According to the multivariate random-effect model, overall effect size was statistically significant (p < 0.0001), estimated as 0.18 (95% CI: [0.13, 0.24]) (Fig. 1). Among the studies with a greater effect size (0.52–0.97), two (Beck et al., 2010; Bigorra et al., 2016a, b) assessed non trained EF with an indirect (teacher or parent questionnaires) measure of everyday executive functioning, and two with a direct measure of attentional control (Bigorra et al., 2016a, b) and switching (de Vries et al., 2015) (Fig. 2).

#### Clinical Symptoms

**Fig. 3:**
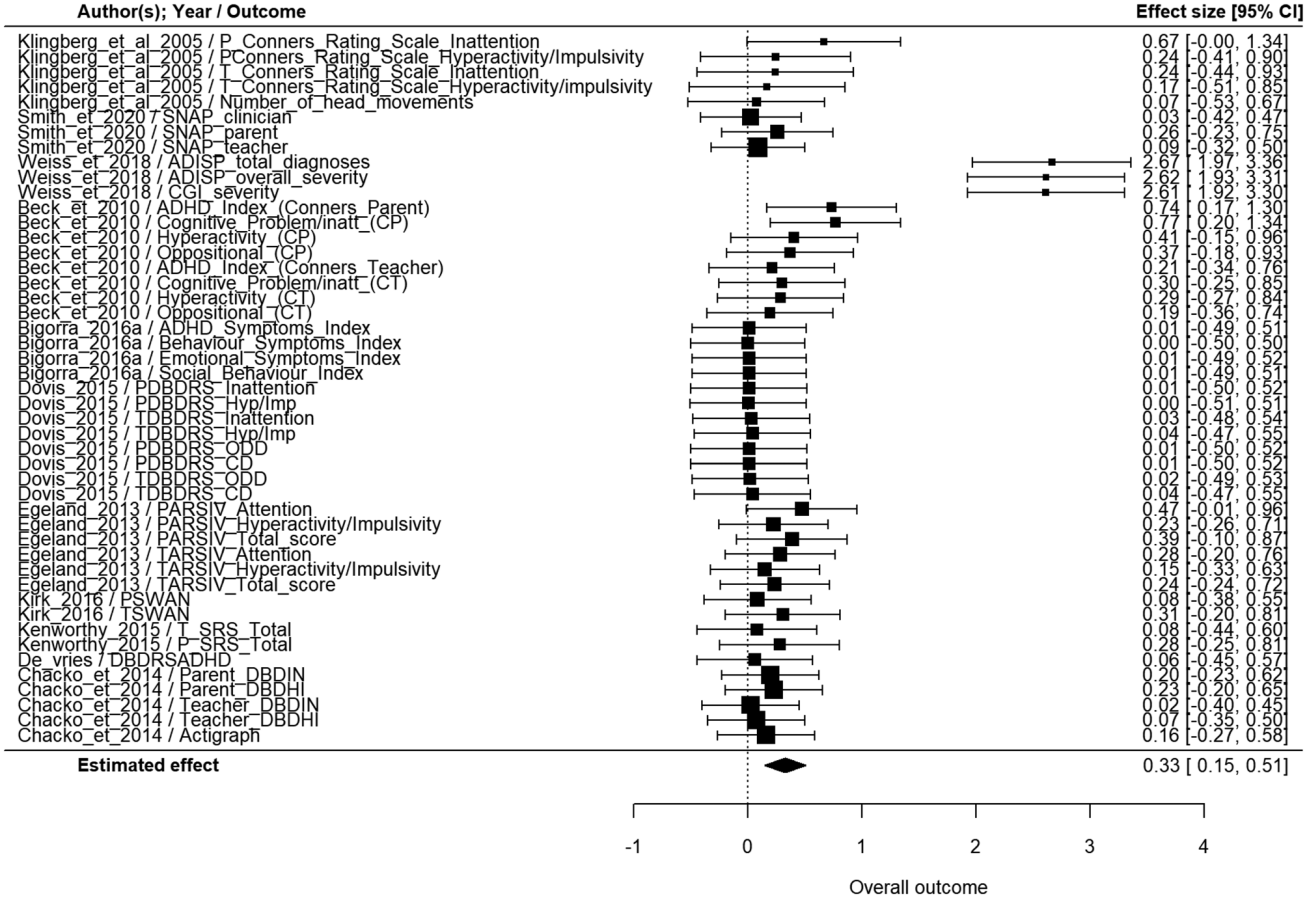
Metanalysis results of far effect on clinical symptoms

Among the 13 studies that assessed clinical symptoms as the far effect, only those with a control group were included. For this reason, two studies were excluded (Leins et al., 2007; Strehl et al., 2017). Other measures included in some studies (Smith et al., 2020; Weiss et al., 2018) have been excluded because of zero sample variance. According to the multivariate random-effect model, overall effect size was statistically significant (p < 0.001), estimated as 0.33 (95% CI: [0.15, 0.51]) (Fig. 2). Among the studies with a greater effect size (0.67–2.67), two considered ADHD symptoms (Beck et al., 2010; Klingberg et al., 2005), assessed with standardized questionnaires, while the other one considered ASD symptoms (Weiss et al., 2018) assessed through an interview conducted with parents by clinician and with a blind clinical global impression (Fig. 3).

#### Learning Skills

**Fig. 4:**
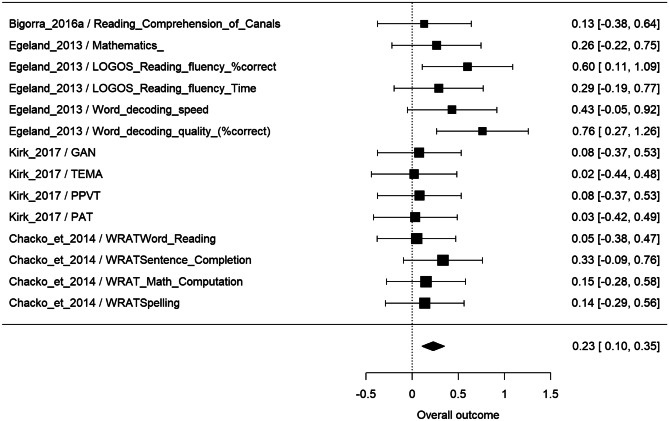
Metanalysis results of far effect on learning skills

All of the 4 studies that assessed learning as a far effect were included in the metanalysis, considering 14 outcome measures. According to the multivariate random-effect model, overall effect size was statistically significant (p < 0.001), estimated as 0.23 (95% CI: [0.10, 0.35]) (Fig. 4). The only study that found greater effect sizes (0.60–0.76) evaluated reading accuracy (Egeland et al., 2013) in ADHD children. (Fig. 4).

#### Cognitive Outcomes

**Fig. 5:**
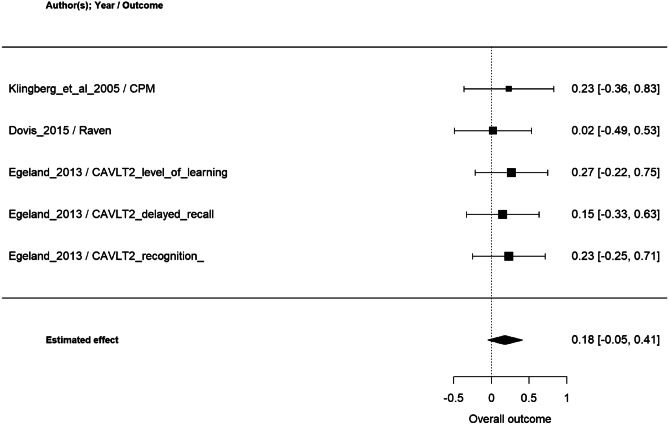
Metanalysis results of far effect on cognitive measures

Among the 5studies that assessed cognitive outcomes as far effects, only those with a control group were included. For this reason, two studies were excluded (Leins et al., 2007; Strehl et al., 2017). According to the multivariate random-effect model, overall effect size was not statistically significant, estimated as 0.18 (95% CI: [-0.05, 0.41]) (Fig. 5).

#### Daily Life Functioning

**Fig. 6:**
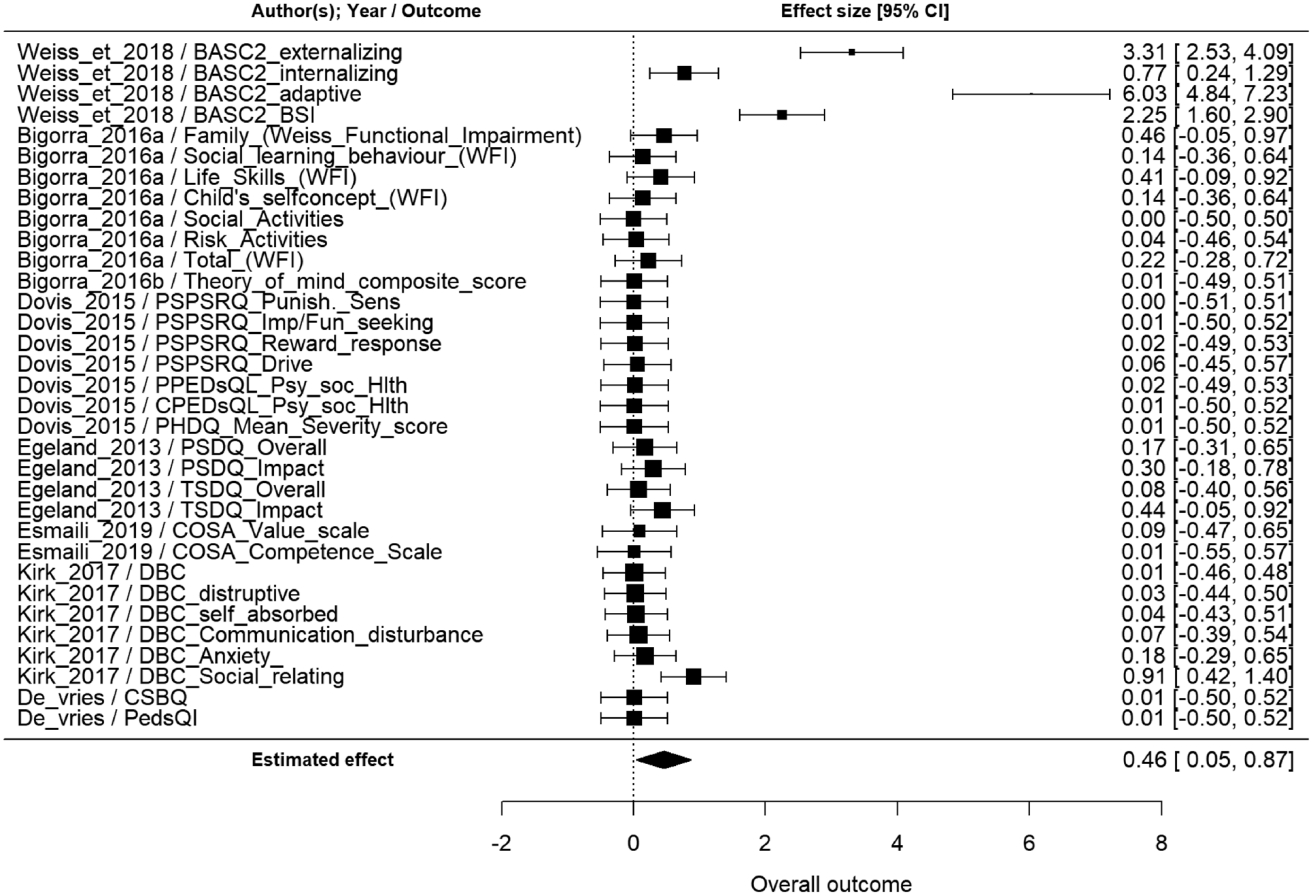
Metanalysis results of far effect on daily life functioning

Among the 10 studies that assessed daily life functioning as far effect, only those with a control group were included. For this reason, two studies were excluded (Leins et al., 2007; Strehl et al., 2017). One study had been excluded because of zero sample variance (Bowling et al., 2017). According to the multivariate random-effect model, overall effect size was statistically significant (p < 0.05), estimated as 0.46 (95% CI: [0.05, 0.87]) (Fig. 6). Among the studies with a greater effect size (0.91–6.03), one (Weiss et al., 2018) investigated behavioural and emotional functioning through a parent report questionnaire in children with ASD, and the other one (Kirk et al., 2017) assessed social functioning in children with intellectual disability.

## Discussion

This systematic review was aimed at investigating the far-transfer effects, which are improvements on any skills or behaviour not directly trained, following EF intervention in children with neurodevelopmental disorders. In fact, in neurodevelopmental disorders and in atypical developmental trajectories, EF alterations are a common finding, suggesting that an executive dysfunction is a pervasive and shared outcome among different disorders and a transdiagnostic indicator of atypical development (Zelazo, 2020). Nonetheless, these complex, multi-component functions influence other cognitive abilities and, above all, daily life functioning (Marotta & Varvara, 2013; Marzocchi & Valagussa, 2011; Vicari & Di Vara, 2017). According to Zelazo's iterative reprocessing model (Zelazo, 2015) which defines a continuous reciprocal relationship between EFs and cognitive development, it is highly probable that a bidirectional relationship is frequently triggered between the specific alterations of a certain disorder and those of EFs. Alternatively (Lahey et al., 2017), EFs could represent either a cognitive factor that contributes to the aetiology of the disorder or a causal factor for the emergence of additional symptoms, making the disorder more complex and severe. Therefore, EF intervention should ultimately improve non trained abilities as well as induce positive cascade effects on development.

Among the different definitions of far transfer (Diamond & Ling, 2016; Klahr & Chen, 2011), for the purpose of this review all the skills not directly involved in the EF intervention and assessed post-intervention have been considered (Melby-Lervåg & Hulme, 2013; Sala & Gobet, 2016, 2017). This conceptualization is in line with the one proposed by Borella and Carretti (Borella & Carretti, 2020), who define as "near transfer" the improvement in the trained skill measured with different tests and "far transfer" the effective generalization of the training effects to tests that detect skills or processes other than those trained. This conceptualization was also used to include articles that did not refer explicitly to "far effect" or "far transfer" in order to provide a more comprehensive overview with respect to the cross-functional effects of interventions on EFs among neurodevelopmental disorders. This approach was used to weigh the impact that improvements in executive functioning have on symptoms or weaknesses characterizing a specific developmental disorder.

According to the Prisma method, out of 1683 studies, only 17 studies met the inclusion criteria. All the studies included, except one (Beck et al., 2010), were randomized control trials, where at least one experimental group and one control group were involved, supporting the quality of the studies according to the National Health and Medical Research Council (NHMRC) Evidence Hierarchy (NHMRC, 2009). Among these, 10 studies reported an improvement right after the intervention in at least one outcome that can be considered as a far effect following an EF treatment.

The results can be summarized by subdividing them according to the main EF components targeted by the interventions.

Among the three studies on *attentional control and inhibition* only one study demonstrated at least one far effect (Kirk et al., 2017). With regard to the interventions on *working memory*, four out of seven studies proved to be effective in producing at least one far effect (Beck et al., 2010; Bigorra et al., 2016a, b; Egeland et al., 2013; Klingberg et al., 2005), while the only study on *cognitive flexibility* intervention did not show any far effect. Thus, interventions on *cold EFs* show high variability on the results: although there is a prevalence of far effects in studies of working memory training, one should note that these prevail in number with respect to those training other EF components. Such a prevalence could be due in part to the exponential increase of interventions on working memory implemented through computerized trainings, that also payed attention to measuring far effects. In contrast, all the three studies on *hot executive* function intervention reported at least one far effect (Bowling et al., 2017; Strehl et al., 2017; Weiss et al., 2018). Finally, among the four studies on *integrated* interventions on different EF components, two reported at least one far effect (Dovis et al., 2015; Kenworthy et al., 2014).

Albeit few in number, interventions on “hot” components of EFs seem promising, probably since the target of the intervention, that is emotional-behavioural self-regulation, appears to be more transversal to a wide range of skills and processes.

With regards to the intervention population, the majority of the studies involved children with Attention Deficit/Hyperactivity Disorder (ADHD), followed by children with Autism Spectrum Disorder (ASD), Intellectual Disability (ID) and Specific Learning Disabilities (SLD). One study conducted an intervention on a population with complex diagnosis, called Behavioural Health Disorders, a mixed category that includes Mood Disorder and ADHD. No studies investigating the far-transfer effects following an EF intervention in children with Developmental Coordination Disorder or with Language Disorder were found. The studies that found at least one far effect were found to be six out of ten for ADHD (Beck et al., 2010; Bigorra et al., 2016a, b; Dovis et al., 2015; Egeland et al., 2013; Klingberg et al., 2005; Strehl et al., 2017), two out of three for ASD ( Kenworthy et al., 2014; Weiss et al., 2018), one out of two in ID ( Kirk et al., 2017), zero out of one in SLD and one out of one in BHD (Bowling et al., 2017). Given the scarce number of studies for each clinical population, conclusive data about the different far effects of EF interventions in different developmental disorders are not obtainable. The preponderance of studies in ADHD might be linked to the hypothesis that EFs are predominantly altered in this neurodevelopmental disorder and extend to different contexts, in part justifying the higher number of far effects respect to other clinical populations.

This review underlines the increasing interest for analysing the impact that intervening on different components of EFs may have on a variety of skills impaired in neurodevelopmental disorders. Thus, such interventions, especially if implemented early on, may indirectly strengthen those functions that become the core deficits or positively shape their developmental trajectories.

As far as the intervention population’s age, all studies targeted school-aged children and three of them expanded the sample to include preschool-aged children. Kirk et al., 2016, 2017; Smith et al., 2020). Among the studies on school-age children, 9 out of 14 found at least one far effect (Beck et al., 2010; Bigorra et al., 2016a, b; Bowling et al., 2017; Dovis et al., 2015; Egeland et al., 2013; Kenworthy et al., 2014; Klingberg et al., 2005; Strehl et al., 2017; Weiss et al., 2018). In the studies including also preschool children, in line with the developmental trajectories of EFs (Lee et al., 2013; Miller et al., 2012; Usai et al., 2014), the proposed interventions targeted the firsts EF component that develops or adopted an integrated intervention perspective, without differentiation of the components, which occurs in later life (Diamond, 2013; Lee et al., 2013; Lehto et al., 2003; Lunt et al., 2012). Among these, only one demonstrated at least one far effect (Kirk et al., 2017).

There was a high variability in frequency, duration and in the EF component target of the intervention. Among the types of EF interventions, computer training activities were the most popular treatments, followed by neurofeedback, interventions embedded in school curricula, individualized manualized Cognitive Behavioural Therapy (CBT) intervention, social activities and physical activities. The following intervention were associated with at least one far effect: computerized training, six out of ten studies (Beck et al., 2010; Bigorra et al., 2016a, b; Dovis et al., 2015; Egeland et al., 2013; Kirk et al., 2017; Klingberg et al., 2005), neurofeedback and curriculum interventions one out of two (Strehl et al., 2017) and manualized CBT ( Kenworthy et al., 2014), and physical activities (Weiss et al., 2018) one out of one for and (Bowling et al., 2017) interventions, while the study that carried out an intervention including social activities did not find any far effect. The results show high variability in interventions examined and in the number of studies for each type of training, not allowing to define whether it is the type of intervention or other characteristics of it that make it effective in determining far effects.

The duration of the interventions varied from 5 weeks to three months with a minimum frequency of 2 times a week and a maximum of every day. Among the ten studies that reported at least one far effect after EF training, 6 reported an intensive and high frequency weekly intervention plan (5 times a week, from 5 to 7 weeks), with sessions of short duration (20—40 min) (Beck et al., 2010; Bigorra et al., 2016a, b; Dovis et al., 2015; Egeland et al., 2013; Kirk et al., 2017; Klingberg et al., 2005), while the remaining studies report heterogeneous data on the frequency, intensity and duration of the interventions. These results extend previous literature by suggesting that frequent and intensive intervention have greater efficacy (Diamond & Ling, 2016) also in terms of far effects.

Concerning the definition of far effects, this review underlies the heterogeneity of the meaning of this term. In fact, “far effect” appears to be an umbrella term that includes different degrees of remoteness from the target of the intervention. Extreme variability was found in the far effect-outcome measures chosen by the various studies that ranged from EFs other than those trained, clinical symptoms, child's daily life functioning, learning skills and other cognitive functions. This variability is partly linked to the different scopes of the studies, the different populations involved but also to the absence of a consensus on definition of far-transfer effect in the literature and the lack of data on the effective utility of implementing an EF training to benefit other skills impaired in different neurodevelopmental disorders. For this reason, a metanalysis was conducted, in order to quantify the effect of EF trainings on each outcome measure of far effects. Among the 17 studies included in the systematic review, only those with a control group were considered for the metanalysis. For the cognitive outcome measures none of the studies found significant effect sizes, demonstrating that executive function interventions are unable to actually produce changes in cognitive functioning measures. The results, in general, are difficult to interpret, due to the very large ICs that reveal small and inaccurate overall effects. These issues also occur with respect to far transfer with greater effect sizes, i.e. daily life skills and clinical symptoms. For these reasons is difficult to draw clear conclusions from the metanalysis about which far transfer effect is more significant than others.

This review underlines the importance of considering the specific disorder’s symptomatology or area of functional weakness as a far effect, in order to clarify which interventions on EFs are preferable (as more effective) for specific clinical population and treatment needs. Considering this interpretation, which underlines the importance of the specific difficulties of each disorder within the context of daily life in the choice of a treatment, it is possible to re-examine the results, which have been described above according to the components of EF target intervention. Six studies on ADHD, which is the population most represented in the literature, have shown significant effects on clinical symptoms or areas of weakness detected by questionnaires (Beck et al., 2010; Bigorra et al., 2016a, b; Klingberg et al., 2005; Strehl et al., 2017), and direct assessments (Bigorra et al., 2016a, b; Dovis et al., 2015; Klingberg et al., 2005). Among the studies that reported an effect on clinical symptoms or areas of weakness assessed directly or indirectly, five utilized computerized intervention programs aimed at enhancing working memory, in school-age groups (7–19 years), for a total duration of 5–6 weeks and with high intensity (from a minimum of 5 times a week to every day).

Among the fewer studies investigating far effects in other neurodevelopmental disorders, only two reported reduction in symptomatology (Kirk et al., 2017; Weiss et al., 2018).

The first used an intensive (20 min per day, 5 times a week for 5 week) computerized treatment targeting various attentive dimensions and learning initiatives in patients with ID. The second involved a 10–14 weeks home/school-based group treatment program on social skills and emotional regulation in patients with ASD.

In an effort to synthetise this results, computer-based treatments are the most studied interventions and seem to be promising for inducing significant far effects in terms of improvement of symptoms and areas of weaknesses. This may be probably due to the characteristics of auto-adaptivity that allows for activities to be always calibrated to one's own performance so as to be challenging for one’s own skills (Klingberg et al., 2005; Thorell et al., 2009), and to the characteristics of enjoyability, which through gamification increases the motivation and fun experienced by the child who performs them (Piqueras et al., 2013; Saine et al., 2011; Torgesen et al., 2010). Furthermore, another feature that could increase the effectiveness of these interventions, also shared by another intervention that has shown significant effects (Weiss et al., 2018), is that it is totally or partially home-based. Although no direct comparisons have been conducted, this feature probably allows for greater intensity of treatment and for embedding the intervention in the context of daily life, actively engaging caregivers.

## Conclusion

Drawing definitive conclusions from this analysis on far effects after EF treatments in children with neurodevelopmental disorders is still very complex. A conceptualization of far effect across different neurodevelopmental disorders was needed. A broad definition of “far transfer effect”, was adopted to include all the skills not directly involved in the EF intervention and focusing the impact on symptoms or weaknesses characterizing a specific neurodevelopmental disorder.

It is necessary to consider the high disparity in the representation of these disorders in this field of study. A higher number of far transfer effects in ADHD maybe in part due to the predominance of intervention studies in this population, in the face of less availability of data relating to other neurodevelopmental disorders, in which, however, this review documents far effects as well. This heterogeneity is also present with regard to the type of treatment on EFs, with a greater representation of studies that analyse the effects of computerized training, probably in line with the increase in computer-based treatment programs for EFs, which have spread over the last decade and proved highly effective in the treatment of directly treated EF components. Nevertheless, different types of interventions analysed may produce far effects. Beyond the type of intervention, intensity, frequency and the possibility of being embedded in daily life contexts, actively engaging caregivers, seem to be the most influential variables in determining far effects. From a practical standpoint, however, an intervention with these characteristics could be scarcely feasible in the traditional taking in charge, requiring significant resources in terms of time and costs, as well as the involvement of the family system.

The current review has some limitations. First, it is important to take into account that not all the studies included use the terms *far effect* or *far transfer* to refer to effects other than those on target functions. This uncertainty about the terminology prompted the authors of this systematic review to select a definition of far effect on the basis of the available literature that appeared most suitable in the context of the study of neurodevelopmental disorders. Some articles, despite having studied the far effects of EF interventions, could have used different terminologies than those used in this review as keywords may have escaped the search. Another noticeable limitation derives from a characteristic inherent in the construct of EFs, the task impurity, for which we cannot exclude that some tasks used to evaluate the far effects in terms of non-targeted EF actually require the involvement of some transversal executive processes directly treated or indirectly affected by the intervention. Overall, this review pays the cost of heterogeneity at the level of population, type of intervention, far effects analysed. This limit made the meta-analysis work complex, as it was necessary to consider the heterogeneity of all the different aspects investigated. We have tried to account for this in our work, directing readers to multiple possibilities of interpretation, underlining however the need to standardize the scientific language and follow common methods in collecting data and setting up future research. Our meta-analysis managed to take into account some of the prescribed recommendations to increase the reproducibility of meta-analyses (Lakens et al., 2016), such as the involvement and direct support of statistical experts, adherence to the PRISMA paradigm, the most detailed disclosure of meta-analytic data specifying their interpretation. For future meta-analyses in the field to be even more informative, it is important that future studies adhere to a common roadmap in data collection and research designs to facilitate the interpretation and reproducibility of meta-analytic studies.

In spite of the limits mentioned above, a first step in highlighting the need to measure far effects of EF trainings in neurodevelopmental disorders has been accomplished. This review paves the way to future studies about far effects of interventions on EFs in different neurodevelopmental disorders and in different age groups, taking into account the developmental trajectory of EFs and focusing on clinical symptoms and / or areas of weakness specific for each disorder as far effects. This will allow the selection of the most appropriate treatments not only on the basis of the specific EF component targeted by the intervention, but also according to the specific impact on the functional weakness of the disorder. This review may have both clinical and methodological implications. It stimulates greater attention to the far effect induced by the EF treatment on the symptomatology, thus defining more realistic expectations on treatment improvements. The analysis of the features shared by the different types of trainings able to produce far effects also opens the way for a clearer definition of an evidence-based methodology in the EF interventions.

## Data Availability

Authors confirms that all data generated or analysed during this study are included in this manuscript.

## Appendix 1

The complete search string was: “executive function ∗ ” (searched in Title and Abstract) OR attention* OR “working memory” OR “updating” OR “inhibitory control” OR “self-regulation” OR “self-regulation” OR “cognitive flexibility” OR “mental flexibility” OR “shifting” OR “set shifting” OR “effortful control” OR “cognitive control” OR “problem solving” OR “planning” OR “executive control” OR “metacognition” OR “behavioral control” OR “self-control” OR “response inhibition” OR “interference control” OR “executive attention” OR “focused attention” OR “selective attention” (searched in Full Text).

AND (child OR children OR "0–18 years") searched in Full Text.

AND (“neurodevelopmental disorder*” OR “learning disorders” OR “developmental coordination disorder” OR “language disorders” OR “Autism spectrum disorders” OR “attention deficit hyperactivity disorder” OR “intellectual disabilities”) searched in Title and Abstract.

AND (training OR education OR treat* OR rehabilitat* OR program OR improv* OR brain training OR curricul* OR empower* OR therapy OR intervention* OR treatment)) searched in Title and Abstract.

AND (“Far effects” OR “quality of life” OR “learning skills” OR “academic skills” OR “math ability” OR literacy OR comprehension OR reading OR writing OR numeracy OR “self-regulation” OR “emotional-regulation” OR “far transfer” OR “social cognition” OR “school readiness” OR “self-efficacy” OR behavior OR success OR health OR skills) searched in Full Text.

NOT (adult OR adulthood) searched in Title and Abstract.
